# Standard procedures for blood withdrawal in conscious male rats induce stress and profoundly affect glucose tolerance and secretion of glucoregulatory hormones

**DOI:** 10.1016/j.molmet.2023.101689

**Published:** 2023-02-04

**Authors:** Kent Pedersen, Helle Andersen, Christian Fledelius, Jens Juul Holst, Sara Toftegaard Hjuler, Rune Ehrenreich Kuhre

**Affiliations:** 1 Integrated Physiology Research, Department of Obesity and NASH Pharmacology, Global Drug Discovery, Novo Nordisk A/S, Måløv, Denmark; 2 Integrated Physiology Research, Department of Diabetes Pharmacology, Global Drug Discovery, Novo Nordisk A/S, Måløv, Denmark; 3 Department of Biomedical Sciences, Faculty of Health and Medical Sciences, University of Copenhagen, Copenhagen, Denmark; 4 Novo Nordisk Foundation Center for Basic Metabolic Research, Faculty of Health and Medical Sciences, University of Copenhagen, Copenhagen, Denmark

**Keywords:** Glucose intolerance, Handheld blood sampling, Insulin secretion, Incretin hormones, Stress hormones, Rats

## Abstract

**Objective:**

A fundamental difference between physiological and pharmacological studies in rats and humans is that withdrawal of blood from conscious rats necessitates restraint which inevitably inflicts a higher level of stress. We investigated the impact of handling on acute glucose regulation and secretion of glucoregulatory hormones in rats.

**Methods:**

Fasted male Sprague Dawley rats (375–400 g, n = 11) were given an oral glucose tolerance test (OGTT) by gavage (2 g/kg). Blood was sampled frequently until 90 min after challenge by handheld sampling (HS) or by automated sampling (AS). In the HS experiment, blood was withdrawn by restraint and sublingual vein puncture; two weeks later, samples were obtained by AS through an implanted catheter in a carotid artery, allowing sampling without disturbing the animals.

**Results:**

On the day of HS, post challenge glucose AUCs were ∼17% higher (P < 0.0001), despite gastric emptying (AUC) being reduced by ∼30% (P < 0.0001). Plasma insulin AUC was 3.5-fold lower (P < 0.001), and glucose-dependent insulinotropic peptide (GIP) AUC was reduced by ∼36% but glucagon-like peptide-1 concentrations were not affected. Glucagon concentrations were higher both before and after challenge (fold difference in AUCs = 3.3). Adrenocorticotropin (ACTH) and corticosterone AUCs were 2.4-fold and 3.6-fold higher (P < 0.001), respectively.

**Discussion and conclusion:**

Our study highlights that sampling of blood from conscious rats by sublingual vein puncture inflicts stress which reduces glucose absorption and glucose tolerance and blunts secretion of insulin and GIP. As blood sampling in humans are less stressful, standard procedures of conducting OGTT's in rats by HS presumably introduce an interspecies difference that may have negative consequences for translatability of test results.

## Highlights

• Blood sampling in conscious rats often requires restraint, inducing stress response.
• Handheld blood sampling inhibits gastric emptying, delaying glucose absorption.
• Handheld sampling impairs glucose tolerance after an OGTT.
• Handheld sampling blunts insulin and enhances glucagon responses after an OGTT.
• Stress-related blunting of OGTT results may complicate rat-to-man translatability.

## 1 Introduction

Mice and rats are the most frequently used animal models in metabolic research [1]. For acute studies investigating blood glucose regulation and secretion of glucoregulatory hormones, rats are preferred due to their higher blood volume. In contrast to voluntary blood sampling in humans, blood sampling in conscious rats normally necessitates restraint which inevitably inflicts stress. Furthermore, blood sampling, most often from either a tail vein, jugular vein or from a sublingual vein, is more invasive than sampling in humans from an indwelling forearm catheter. Other non-terminal sampling procedures of venous blood includes sampling from saphenous vein or dorsal pedal vein [2], which would also be anticipated to inflict a higher level of stress than that occurring with humans. Level of stress during glucose tolerance tests may, therefore, constitute an underappreciated inter-species factor that might influence the translatability of data from rodents to humans. The aspect is, however, understudied – presumably because blood sampling from rats without handling is difficult. To investigate the impact of handheld blood sampling (HS) on acute glucose regulation and secretion of glucoregulatory and stress hormones after an oral glucose tolerance (OGTT), we sampled blood from the same rats using two different procedures: by restraining and sublingual vein puncture (HS) and by automatic blood sampling (AS) through an implanted catheter in the common carotid artery, allowing sampling without handling. In both cases, rats were conscious during the experiment. Alternatively, the rats could have been studied during anaesthesia, which presumably would have reduced stress resulting from gavage and blood sampling. However, as anaesthesia dramatically influence gastrointestinal motility, oral glucose tolerance, and plasma insulin responses to an OGTT [3], this approach would presumably have confounded data more than the stress-response in conscious rats.

## 2 Material and methods

### 2.1 Ethical considerations

Animal studies were conducted with permission from the Danish Animal Experiments Inspectorate (2020-15-0201-00683) in accordance the National Institutes of Health (publication number 85-23) and the European Convention for the Protection of Vertebrate Animals used for Experimental and other Scientific Purposes (Council of Europe No 123, Strasbourg 1985).

### 2.2 Animals, housing, and pre-experimental procedures

Normal weight Sprague Dawley male rats (6 weeks of age) were purchased from Janvier (Saint Berthevin Cedex, France). Rats were acclimatized for 2 weeks, following a 12:12 h light:dark cycle with lights on at 0600 h and with ad libitum access to chow (Altromin 1324). After acclimatization, rats were transferred to Accusampler® cages (n = 1/cage) and left for two weeks, following the same light:dark and food regimen. A week after, rats were mock handled (restrain in neck and back skin and tong-lifting with a needle) for 2 days to reduce handling-induced stress responses at day of OGTT. The following day, rats were fasted for 6 h, were restrained and had an OGTT performed by gavage (2 g glucose/kg body weight). The glucose solution contained in addition paracetamol (100 mg/kg). Blood was withdrawn by sublingual vein puncture (400 μL/sample) and transferred to pre-chilled EDTA coated tubes which were instantly put on ice and centrifuged (2400×*g*, 10 min, 4 °C) within 15 min and were stored at −20 °C. Sampling timepoints were: -10 min (baseline, plotted as 0 min), 5, 15, 45, 60, and 90 min. Glucose was dosed at 0 min. After the OGTT, rats were left undisturbed for two weeks. Hereafter, rats were anaesthetised with isoflurane and had a permanent catheter, filled with heparinized saline, inserted into a common carotid artery with the tip advance to the thoracic aorta. The catheter was led subcutaneously to the dorsal region of the neck and were fixed by use of a Covance Infusion Harnesses™ and immediately connected to automatic blood sampling equipment (Accusampler®) for catheter maintenance. Rats were left connected for four days prior to the OGTT for adaptation. After the operation, rats were dosed with enrofloxacin (Baytril® 10 mg/kg s.c.), buprenorphine (Temgesic® 0.05 mg/kg s.c.) and carprofen (Rimadyl ®Vet. 5 mg/kg s.c.). Carprofen treatment was repeated 24 h after. After 4 days recovery, the OGTT procedure described above was repeated, but in this case, blood was sampled automatically through the indwelled catheter from freely moving undisturbed rats.

### 2.3 Biochemical analysis

Plasma concentrations of glucose and acetaminophen were measured on a Cobas 6000 analyzer (Roche, Basel, Switzerland) (Cat. no.4404483190 and 06769942190). Plasma concentrations of glucoregulatory hormones were quantified by ELISA's from Mercodia (Uppsalla, Sweden): glucagon (Cat. No. 10-1281-01, glucagon-like peptide-1 (total) (Cat. No. 10-1278-01) and insulin (Cat. No. 10-1250-01) or from other vendors: ACTH (Cat. No. ab263880, Abchem, Cambridge, UK), corticosterone (Cat. No. EIACORT, Invitrogen, Thermo Fisher Scientific, MA, USA), and glucose-dependent insulinotropic peptide (GIP) (Cat. No. EZRMGIP-55K, Sigma Aldrich, Merck Life Science A/S, Søborg, Denmark).

### 2.4 Data presentation and statistical analysis

Data are shown as means ± SEM. Graphs were made in GraphPad Prism 9 (La Jolla, CA) and figures were edited in Adobe Illustrator (Adobe Systems Incorporated, San Jose, CA). Statistical significance was assessed using GraphPad Prism. The applied tests are indicated in figure legends. P < 0.05 was considered statistically significant. AUCs presented are total AUCs with a baseline set to 0.

## 3 Results

### 3.1 HS collection of blood increases plasma concentrations of adrenocorticotropic hormone and corticosterone

**Figure 1:**
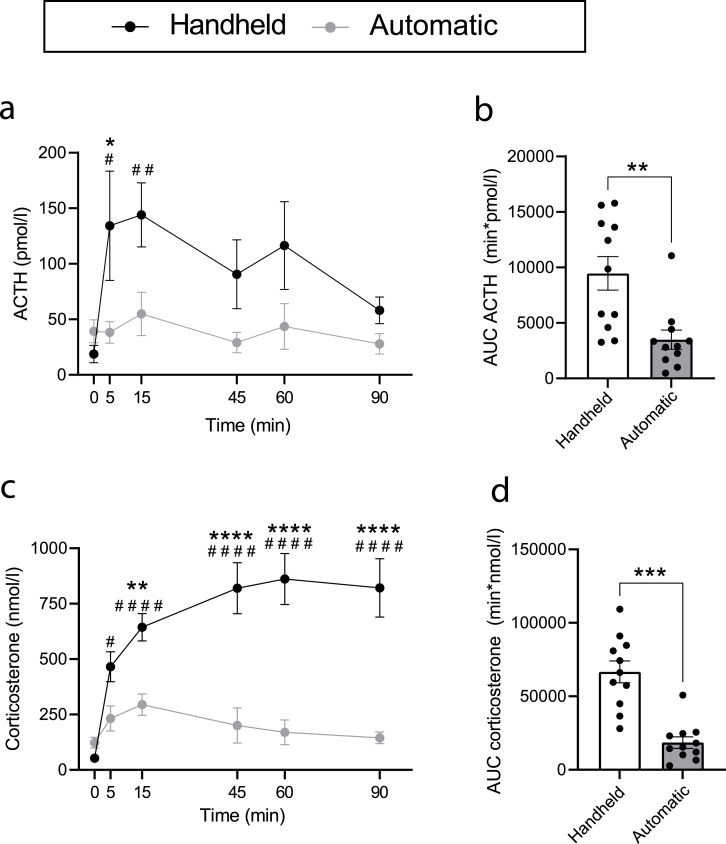
**Handheld blood sampling increases circulating concentrations of stress hormones.** A: Plasma ACTH (pmol/l), B: AUC plasma ACTH (min∗pmol/l), C: Plasma corticosterone (nmol/l), and D: AUC plasma corticosterone (min∗(nmol/l)). Statistical significance was tested by two-way ANOVA for repeated measurements followed by Sidak multiple comparison test (A and C) or by paired *t* test (B, and D). ∗∗∗P < 0.001, ∗∗∗∗P < 0.0001 between study days; ^#^P < 0.05, ^####^P < 0.0001 between respective baseline and indicated time points; black: handheld sampled, grey: automatically sampled. Dots in bar graphs represent different animals. Data are shown as means ± SEM.

To indirectly investigate the impact of HS on stress level, we measured the two stress hormones corticosterone and adrenocorticotropic hormone (ACTH)) after HS and AS. Plasma concentrations of ACTH did not differ at baseline, but rapidly increased after the OGTT on the HS day, but not on the day of AS. AUCs differed 2.4-fold (Figure 1A,B). Corticosterone concentrations did not differ at baseline (P = 0.99), but after glucose challenge and until end of sampling, concentrations were, consistent with the ACTH data, higher at the day of HS and the AUC was 3.6 fold greater (P < 0.001, Figure 1C,D).

### 3.2 Handheld blood sampling impairs glucose tolerance and prolongs gastric emptying

**Figure 2:**
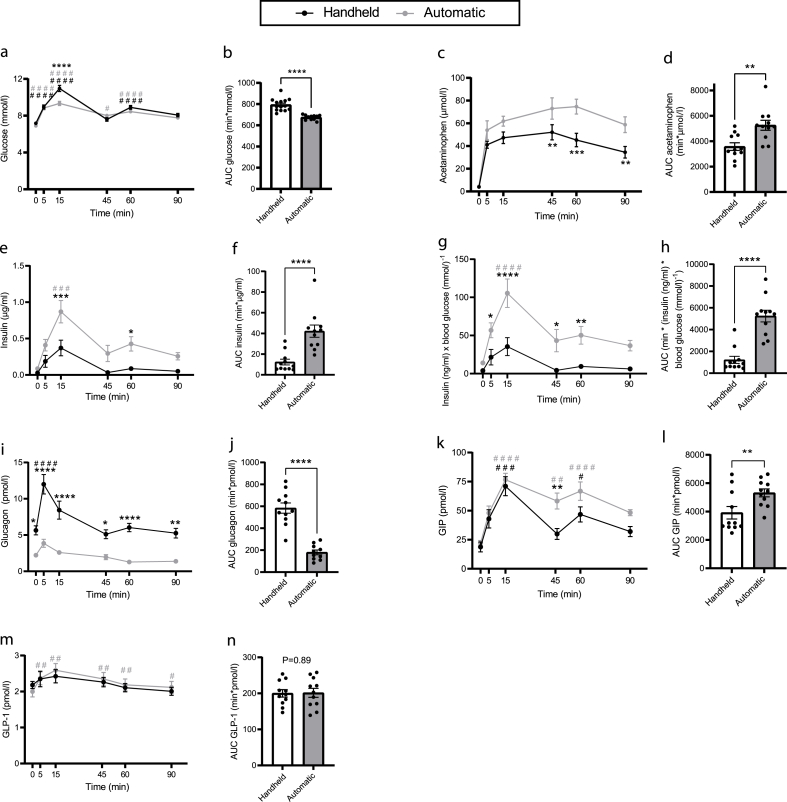
**Handheld blood sampling reduces glucose tolerance, prolongs gastric emptying and blunt the insulin and incretin response to oral glucose challenge.** A: Plasma glucose (mmol/l), B: AUC plasma glucose (min∗mmol/l), C: plasma acetaminophen (μmol/l), D: AUC plasma acetaminophen (min∗(μmol/l)), E: plasma insulin (μg/ml), F: AUC plasma insulin (min∗(μg/ml)), G: plasma insulin:glucose ratio (insulin (ng/ml)/blood glucose (mmol/l), H: AUC plasma insulin:glucose ratio (min∗(insulin (ng/ml)∗glucose (mmol/l), I: plasma glucagon (pmol/l), j: AUC plasma glucagon (min∗pmol/l). Statistical significance was tested by two-way ANOVA for repeated measurements followed by Sidak multiple comparison test (A, C, E, G, and I) or by paired *t* test (B, D, F, H, and J). ∗P < 0.05, ∗∗P < 0.01, ∗∗∗P < 0.001, ∗∗∗∗P < 0.0001. ^#^P < 0.05, ^##^P < 0.01, ^###^P < 0.001, ^####^P < 0.0001 between respective baseline and indicated time points; black: handheld sampled, grey: automatically sampled. Dots in bar graphs represent different animals. Data are shown as means ± SEM.

Fasting blood glucose concentrations were not different at baseline (P = 0.91, Figure 2A). Post glucose AUCs were ∼17% higher at HS (Figure 2B), although plasma acetaminophen concentrations (marker of gastric ventricle emptying [4]) were reduced by ∼30% (AUC, Figure 2D).

### 3.3 HS collection of blood blunts the insulin and incretin response to oral glucose challenge

Fasting plasma insulin concentrations did not differ between study days, whereas AUC was 3.4-fold higher following AS (Figure 2E,F). Normalised for blood glucose concentrations, the difference was 4.3-fold (Figure 2G,H). In contrast, plasma glucagon concentrations were increased by HS; both before and after challenge (fold difference in AUCs: 3.2, Figure 2I,J). Concentrations of the two incretin hormones, GIP and GLP-1 were similar at baseline (P ≥ 0.96), but GIP AUCs were ∼36% higher at AS (P < 0.01, Figure 2K,L). GLP-1 concentrations were generally low (<4 pmol/l) and AUCs did not differ.

## 4 Discussion

The main finding in our study is that standard HS procedures of blood withdrawal in conscious male rats have considerable impact on the most commonly investigated OGTT endpoints. In particular, despite a pronounced attenuation of the insulin response, as well as pronounced increased plasma glucagon concentration (which presumably increased hepatic glucose output [5]), plasma glucose concentrations were minimally affected. This may be related to the slower and prolonged gastric emptying and a similarly delayed reduced glucose absorption rate [6]. Although not directly investigated in our study, the underlying reason for the attenuated insulin response and increased glucagon response is likely to be diffuse sympathetic discharge and increased circulating corticosterone levels; indeed, both are known to inhibit insulin secretion and stimulate glucagon secretion [7]. On top of this, reduced secretion of GIP may be of importance for the reduced insulin response, since GIP and GLP-1 normally potentiate glucose-stimulated insulin secretion, accounting for up to 75% of the postprandial insulin response [8]. Whether GLP-1 responses to an OGTT are equally affected by blood sampling technique is more diffucult to judge based on our data since the measured GLP-1 (total) concentrations were very low and about 10-fold lower than corresponding levels in humans [9]. Perhaps this inter-species difference in quantified concentrations may be caused by different metabolism of GLP-1 since both intact (7–36 amide) and truncated (9–36 amide) GLP-1 in mice, unlike in humans, are eliminated within a few minutes owing to extensive endoproteolytic cleavage by NEP 24.11 [10], giving rise to underestimation of actual GLP-1 secretion if the assay employed, as in this study, is based on a sandwich ELISA requiring presence of both the N- and C-terminus of the molecule for measurement. An limitation of our study is that blood, for practical reasons and for mimicking the most commonly used techniques in pharmacological research, was collected from a vein on the HS day and from an artery on the AS day. More specifically, the reasoning behind the arterial catheter insertion is that we in previous model development experiments have found that indwelling of the catheter in a vein significantly impaired patency and catheter function (blood withdrawal). We thus judged that arterial insertion would be the more feasible approach, while arterial sampling would not be practical for HS in conscious animals. The detected differences in the measured parameters could, therefore, have been influenced by arteriovenous concentrations differences. However, controlled studies in humans comparing concentrations in venous blood that have been “arterialized” by the “heated-hand-technique” and non-arterialized venous blood indicates that arteriovenous differences after an OGTT are either undetectable or below 20% in case of corticosterone, glucose, glucagon, GLP-1, and insulin [11,12], compared to the >200% differences in our study. Whether same differences would have applied if the glucose challenge had been given intravenously rather than orally is an interesting and relevant question yet to be investigated. First, the intravenous administration would require catheterization or intraperitoneal administration, which should be compared to oral gavage. Secondly, although the intravenous administration would not be sensitive to stress-induced changes in gastric emptying, the stress from HS would still impact e.g. insulin secretion via sympathetic stimulation. Most likely, therefore AS would still be preferable.

A fundamental question regarding the relevance of our study is whether stress responses were reduced at the day of AS. Based on plasma ACTH and corticosterone concentrations, this appears to have been the case, since concentrations at day of AS only increased transiently after the OGTT and were uninfluenced by blood sampling per se. Similarly, plasma cortisol concentrations are uninfluenced by an OGTT and frequent blood sampling in humans [13]. Based on this, the AS method would be expected to have higher translational value than HS. AS is, however, a not suitable as a default experimental model as it is expensive, laborious and have low-through put. AS may, instead, be a valuable model for studies where minimization of stress is of particular importance. For instance, we used AS to investigate whether the stress and appetite inhibiting hormone growth differentiating factor 15 affects the hypothalamic-pituitary-adrenal axis [14].

## Author contributions

KP, HA and REK concepted and designed the study. KP, HP and REK executed experiments and processed and analysed data. REK drafted the manuscript; KP, HP, CF, JJH, and STH revised manuscript and contributed significantly with intellectual content. All authors approved submitted version of manuscript.

## Data Availability

Data will be made available on request.
